# Effects of Forage-to-Concentrate Ratio on Abnormal Stereotypic Behavior in Lambs and Goat Kids

**DOI:** 10.3390/ani15070963

**Published:** 2025-03-27

**Authors:** Cemil Tölü

**Affiliations:** Department of Animal Science, Faculty of Agriculture, Çanakkale Onsekiz Mart University, 17100 Çanakkale, Türkiye; cemiltolu@comu.edu.tr; Tel.: +90-286-218-00-18 (ext. 23072)

**Keywords:** Tahirova, Turkish Saanen, abnormal stereotypic behavior, repetitive grooming, cortisol

## Abstract

A crucial component of small ruminant production, particularly where intensive systems are becoming more prevalent, is determining the forage-to-concentrate (F:C) ratio in their daily diets. The effects of the F:C ratio in lambs and goat kids’ daily diets on growth, stereotypical behavior, and blood biochemical parameters were investigated. Eighteen Tahirova sheep lambs and 18 Turkish Saanen goat kids were used, divided into three groups using the following F:C ratios: 20:80, 60:40, and 80:20. Growth rates, weight gains, and feed conversion ratios were determined. Urea nitrogen, glucose, non-esterified fatty acids, creatine kinase, and cortisol hormones were measured in blood samples. In lambs, growth remained consistent across F:C groups. In goat kids, however, the 20:80 group showed superior growth and weight gain. Based on the F:C ratios, behavioral differences were found in forage feeding, rumination, water consumption, and standing activities. Similarly, the 20:80 group displayed higher rates of bar biting, crib biting, and wool biting in lambs, as well as bar biting, crib biting, bucket biting, and chain chewing in goat kids. Cortisol levels were significantly higher in the 80:20 group than in the 20:80 group for both species. Insufficient forage can cause abnormal stereotypical behaviors, which negatively impact animal welfare.

## Simple Summary

The effects of the forage-to-concentrate (F:C) ratio in lambs and goat kids’ daily diets on growth performance, stereotypical behavior, and blood biochemical parameters were investigated. Animal behaviors and feed and water intake were monitored closely for eight hours a week in individual pens. The normal and abnormal stereotypic behaviors observed in lambs and goat kids were recorded either as ratios in time sampling or in continuous sampling. The results indicate that normal and abnormal stereotypic behaviors varied between the groups. Forage feeding and concentrate feeding behaviors aligned with the F:C ratios, while rumination behavior correlated with the roughage ratio in the diet. Lambs exhibited higher frequencies of bar biting, crib biting, and wool-biting behaviors, whereas goat kids displayed more bar biting, crib biting, bucket biting, and chain chewing behaviors in the 20:80 group than other groups. Comparing normal and abnormal behaviors in lambs and goat kids, it appears that goat kids are more affected by the low forage ratio in their daily diet than lambs. Additionally, repetitive grooming behavior in goat kids might transform into abnormal stereotypic behavior, raising concerns about animal welfare.

## 1. Introduction

Ruminant species sharing similar digestive physiologies exhibit diverse preferences for grasses, legumes, flat-leaved plants, or other plant species [1]. In their natural habitats, these animals spend significant portions of their day engaging in activities such as grazing, chewing, and rumination. However, the shift from extensive to intensive animal production systems has led to a reduction in the feeding time of farm animals, many of which now spend the majority of their days in enclosed shelters. This change has been accompanied by challenges such as limited grazing opportunities, insufficient forage, and issues related to forage quality. Additionally, the pursuit of higher performance and the practice of providing concentrated feed further compound these challenges in intensive ruminant production systems. Consequently, these factors have detrimental effects on the performance, behavior, and overall health of animals [2,3,4].

Carbohydrates are broken down by microorganisms in the rumen into a variety of byproducts, including acetic acid, propionic acid, butyric acid, carbon dioxide, methane, ammonia, and microbial cells. The ratio of forage to concentrate (F:C) in the diet, the animal species, the acetate to propionate ratio, the feed form, and the rumen pH all affect the relative amounts and types of these compounds produced [5,6]. It is speculated that a decrease in rumen pH values occurs when the percentage of concentrated feed in the ration exceeds 60%, while the optimal rumen pH and protozoa fauna exist at a 40–50% forage level [5]. Maintaining an appropriate rumen pH is crucial to protect the rumen from organic acids produced after rumination and to support microbial fermentation. Ruminants manage rumen pH largely through saliva produced during chewing and rumination [7,8].

Daily feeding practices often deviate from the recommended F:C ratio of 60:40, resulting in insufficient consumption based on feed quality. This causes them to react with abnormal, stereotypical behavior [2,9,10,11]. Abnormal stereotypical behavior refers to repetitive, regular, and invariant actions lacking an apparent purpose or function [2,12,13]. Such behavior poses a significant animal welfare concern, resulting in dysfunctional energy consumption, chronic stress, health deterioration, and decreased productivity.

Various animal species demonstrate unique deviant stereotypic tendencies. Captive carnivorous animals housed in zoological facilities exhibit locomotor stereotypic behaviors, while ruminant species, in particular, tend to display oral stereotypic tendencies that deviate from their natural patterns [13]. These behaviors often emerge due to reduced time and energy spent on activities like food search, finding, competition for feeding, chewing and digestion. Factors such as restrictive feeding, decreased feeding time, restrictions in shelter conditions, and husbandry practices contribute to abnormal stereotypical behavior in ruminants [4,13]. Animals may manifest abnormal stereotypic behaviors including bar biting, crib biting, bucket biting, wool biting, pacing, tongue rolling, and floor manipulation [2,9,10,11].

Studies have revealed notable differences in feed intake and live weight values among lambs across various F:C groups [3,14]. Similarly, research on goat kids examined F:C ratios at 30:70, 50:50, and 80:20 leading to variations in parameters such as dry matter intake (DMI), crude protein intake (CPI), total digestible nutrient intake (TDNI), and live weight gain [15]. Assessing diverse nutritional regimes, performance metrics, and both normal and abnormal stereotyped behavior provides comprehensive insights into the animals’ biology. In addition, analyzing stress hormones, blood glucose, creatine kinase (CK), and non-esterified fatty acid (NEFA) levels offers valuable information regarding nutrition, stress, and health conditions in the animal [16].

In this context, the study focuses on Tahirova sheep lambs and Turkish Saanen goats, extensively used breeds in small ruminant milk production. The Tahirova genotype (75% East Friesian, 25% Kıvırcık) was developed using the East Friesian sheep breed in Turkey. The Turkish Saanen breed was developed by crossing the Saanen breed with local goats. Tahirova sheep and Turkish Saanen goats have high milk and litter yields and are important dairy breeds for milk production. In small ruminants, roughage is not used during the growing season, especially in lambs, and generally roughage utilization rates are variable. The research aims to investigate the impact of F:C feeding regimes on both normal and abnormal stereotypic behaviors, representing crucial aspects of animal welfare. The study also assessed the impact of varying F:C ratios in the daily ration on live weight, feed and nutrient intake, specific blood biochemical parameters, and cortisol hormone levels.

## 2. Materials and Methods

The study carried out at Çanakkale Onsekiz Mart University Faculty of Agriculture Farm Animal Production Research and Application Unit (40°07.65′ N, 26°26.38′ E).

### 2.1. Animals and Housing

**Figure 1:**
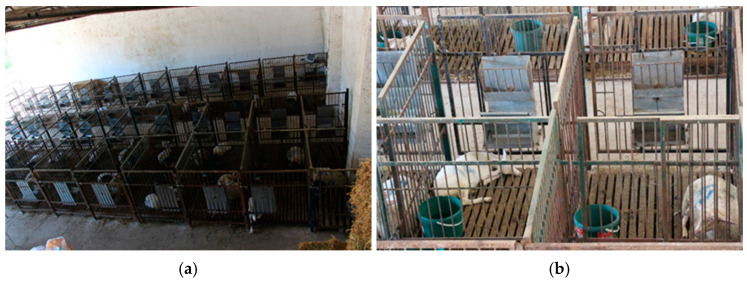
An overview of both the experimental barn (**a**) and individual pens for the lambs and goat kids (**b**).

The study involved 18 female Tahirova sheep lambs averaging 103 days old and 18 female Turkish Saanen goat kids averaging 95 days old. The research spanned 5 weeks and divided the lambs and goat kids into three groups (n = 6) based on the F:C ratio in their daily diets. The animals were evenly distributed into pens according to their species and F:C groups. Group allocation was random, considering the animals’ weights at weaning (60 d old) and their current live weights. Throughout the study, individual pens measuring 1.10 m in width, 1.50 m in length, and 1.50 m in height were used. The pens had concrete floors with wood slated surfaces (5 cm wide, spaced 2 cm apart). The fencing between pens had a gap of 6.5 cm and a thickness of 1 cm. Each pen was equipped with individual feeders and plastic water buckets (Figure 1). Forage was provided in the wire-caged upper section of the feeder, while concentrated feed was placed in the lower section, covered with a lid and chain.

### 2.2. Feeding Management

Prior to the study, all animals underwent a 5-day adaptation phase, during which they were fed the experimental diet. The primary focus of feeding practices for both lambs and goat kids revolved around variations in the F:C ratio within their daily diets. The treatment groups, differentiated based on dry matter intake, were established as 60:40, 20:80, and 80:20 for F:C ratios. According to NRC [17] recommendations, average target weights were determined to be 28 kg for lambs with a daily weight gain of 250–300 g and 15 kg for kids with a daily gain of 150–200 g. Therefore, 1.23 kg, 3.51 Mcal ME, and 152 g CP for lambs and 0.605 kg, 1.95 Mcal ME, and 223 g CP for goat kids were chosen as the target values for daily DMI, daily metabolizable energy (ME), and daily CPI. Alfalfa hay and concentrate feed in pellet form were used for all groups (Table 1). The concentrated feed in pellet form (length 1–2 cm, diameter 0.5 cm) was produced by a commercial feed manufacturer. The feed was characterized as “growth feed”. It contained corn, barley, sunflower meal, soybean meal, DDGS corn, wheat razmol, molasses, calcium carbonate, sodium chloride, ammonium chloride, and vitamin–mineral premix. The alfalfa hay was dried by natural sun in the field at the height of the alfalfa plant (50–70 cm) and packed by a baler into 25 kg bales in the form of rectangular prisms.

Feed and water were given ad libitum twice a day (07:30 and 16:30), and feed consumption and water intake were recorded daily. To ensure cleanliness throughout the day, fresh water was replenished as needed.

### 2.3. Feeding Analyses

The dry matter (DM), ether extract (EE), crude protein (CP), and ash contents of the feeds were analyzed using the methods outlined in AOAC [18] guidelines. Neutral detergent fiber (NDF), acid detergent fiber (ADF), and acid detergent lignin (ADL) content in the feed samples were determined based on the procedures described by Van Soest et al. [19]. To calculate the ME content of the feeds, the formula ME (kcal/kg DM) = 3381.9 − 19.98 × NDF, as established by Kirchgessner et al. [20], was employed.

### 2.4. Performance Measurements

The animals’ initial (LWi) and final (LWf) live weights (LW), as well as weekly weight measurements, were recorded using a precision animal scale accurate to ±20 g before the morning feeding: live weight gain (LW gain in kg, calculated as LWf − LWi), gain rate expressed as a percentage [Gain rate (%) = 100 × (LWf − LWi)/LWi)], and the feed conversion ratio (FCR), calculated as DMI/(LWf − LWi), where DMI represents the total feed intake during the experiment in kg of DM [14]. Additionally, the total nutrient and energy intake per animal per day were calculated based on the 24 h consumption of alfalfa and concentrate feed, taking into account the nutrient composition of the feed.

### 2.5. Behavioral Observations

Weekly and 8 h (08:00 and 16:00) daily behavioral assessments were conducted using two methods: time sampling (at 10 min intervals) and continuous observation. In the time sampling method, the observed behaviors were categorized as either observed (1) or not observed (0), while with the continuous observation method recorded the frequency (number of times) of observed behaviors as they occurred. A team of 6 experienced observers carried out these observations, with each observer assigned to monitor 6 individual pens. During the observations, the observers constantly changed the animals they observed, even during the day. This approach was adopted to mitigate the potential for observer bias. The behaviors observed in lambs and goat kids were recorded either as ratios (in time sampling) or times (in continuous sampling):

Lying: the state where animals are resting without engaging in any other activity (recorded as ratio and times).

Standing: the posture where animals are upright without engaging in any other activity (recorded as ratio).

Forage feeding: the animal’s tendency to bite, eat, and chew alfalfa (recorded as ratio).

Concentrate feeding: the animal’s tendency to bite, eat, and chew concentrate (recorded as ratio).

Rumination: the act of re-chewing cud stored in the rumen for further breakdown, which can occur while the animal is both lying down or standing up (recorded as ratio and times).

Moving: the animal’s activity of moving within the pen (recorded as ratio).

Abnormal stereotypic: any of the defined abnormal stereotypic behaviors monitored in the experiment were observed (recorded as ratio).

Bar biting: chewing and licking the pen bars (recorded as times).

Crib biting: chewing and licking the feeder bars (recorded as times).

Bucket biting: chewing and licking the parts of buckets (recorded as times).

Wool biting: biting and pulling the wool of a neighboring animal with its muzzle (recorded as times).

Chain chewing: chewing and licking the chain on the feeder (recorded as times).

Forage crumbs manipulation: the tendency to engage with spilled forage on the floor, including consuming and chewing (recorded as times).

Pawing: digging the floor with its front legs, excluding lying behavior (recorded as times).

Social contact: the animal’s muzzle being in contact with a neighbor’s pen or body (recorded as times).

Bipedal stance: the animal putting its front foot on the feeder or pen bars (recorded as times).

Grooming/scratching: touching its own body with the tongue or mouth (recorded as times).

Drinking water: the animal’s tendency to approach water and drink (recorded as times).

Oral manipulation behavior was defined as the cumulative instances of bar biting, crib biting, bucket biting, chain chewing, wool biting, and ear biting during observation periods. Since no bedding was present on the floor and spilled forage crumbs were cleared daily, the tendency to interact with these spilled forage remnants was defined as forage crumbs manipulation behavior. The behaviors addressed were modified by taking into account the definitions in previous studies [2,9,10,11].

### 2.6. Blood Chemicals and Hormone Analyses

Blood samples were collected from the *Vena jugularis* before the morning feeding during the initial (first week) and final (last week) stages of the study. After collecting blood samples, they were centrifuged (1643× *g* for 10 min) to separate the serums. The serums were stored in sterile tubes in a deep freezer at −20 °C until the analysis day. Cortisol hormone analysis was conducted on the blood serum using the Enzyme-linked immunosorbent assay (ELISA) method at the Animal Health and Physiology Laboratory of Canakkale Onsekiz Mart University, Department of Animal Science, following the procedures outlined by Tölü et al. [21]. The analyses were conducted using the Thermo Scientific Multiskan FC Microplate Reader (Thermo Fisher Scientific Instruments Co., Shanghai, China), which utilized commercially available hormone kits (Shanghai Sunred Biological Technology Co., Shanghai, China) designed specifically for each animal species. The analyses of biochemical traits and cortisol hormone followed the instructions provided by the relevant kits. The concentrations of blood urea nitrogen (BUN) (IFCC UV) and glucose (GPO-PAP) were determined with a spectrophotometer (UV-mini 1200, Shimadzu, Kyoto, Japan) using a commercial kit (ImproGen Diagnostics Industry, İstanbul, Türkiye). Non-esterified fatty acids (NEFAs) were determined also with a spectrophotometer (UV-mini 1200, Shimadzu, Kyoto, Japan) using an enzymatic kit (Randox Laboratories Industry, Crumlin, County Antrim, UK).

### 2.7. Statistical Analysis

All analysis were performed separately for each animal species. For LW-related analyses, a model (one-way ANOVA) encompassing the F:C ratio effect (20:80, 60:40, 80:20) was employed. Repeated measurements of LW gain and feed consumption values were incorporated into the analysis, accounting for both the F:C ratio and including the control day in the model (MIXED). Blood biochemistry and hormone data were subjected to variance analysis (MIXED), considering the F:C ratio and control day. *Post hoc* analysis for all traits was conducted using the Tukey test.

Behavioral data, obtained through the time sampling method and exhibiting a binomial distribution (0 and 1), underwent analysis using the Generalized Estimating Equations (GEE) method. The model included group (20:80; 60:40, 80:20), observation day (1 to 5), and observation hour (1 to 8) effect. Preliminary statistical analyses indicated the absence of observer effect; consequently, the observer effect was not included in the final analysis. Pairwise contrast based on the Wald Chi-square test was used for significant factors. For continuously observed behavioral data, we first calculated the total number of behaviors per animal and per day. Repeated measures analysis of variance (MIXED) was carried out after logarithmic transformation (y + 10), factoring in F:C ratio and observation day effects. *Post hoc* analysis was conducted using the Tukey test. All statistical analyses were carried out using the SAS [22] software (Version 9.0) package. A *p* value of <0.05 was accepted as the critical value threshold in the analyses.

## 3. Results

### 3.1. Live Weight and Feed Intake

The initial and final live weight (LW) values for both lambs and goat kids within the F:C groups showed no significant differences (Table 2). However, when comparing the final and initial experiment weights, lambs in the 20:80 group exhibited higher LW gains than those in the 80:20 group (*p* ≤ 0.05). In terms of DMI and FCR values, there were no significant differences observed among the F:C groups for both lambs and goat kids (*p* > 0.05). Conversely, significant differences were noted in LW gain and gain rate performance among goat kids based on F:C groups (*p* ≤ 0.0161). Specifically, the 20:80 group displayed higher LW gain and gain rate compared to the other groups (*p* ≤ 0.05).

Table 3 presents water and nutritional intake data for lambs and goat kids based on F:C feed groups. In this study, where alfalfa hay and concentrated feed were provided ad libitum, the F:C ratios in the groups aligned with the intended values. The study revealed notable variations in the consumption of various nutrients, including total organic matter intake (OMI), total CPI, total ether extract intake (EEI), total crude fiber intake (CFI), total neutral detergent fiber intake (NDFI), total acid detergent fiber intake (ADFI), and total metabolizable energy intake (TEI), across different F:C groups in lambs (*p* ≤ 0.05). Specifically, OMI was higher in the 20:80 group compared to the other groups (*p* ≤ 0.05). EEI and TEI values followed the order of 20:80, 60:40, and 80:20, with all groups showing significant differences from one another (*p* ≤ 0.05). Similarly, CFI, NDFI, and ADFI values were ranked as 80:20, 60:40, and 20:80, and significant differences were observed among all groups (*p* ≤ 0.05).

Goat kids exhibited significant differences in nutrient intake across F:C groups, as indicated in Table 3. Notably, the 80:20 group consumed less water compared to the other groups (*p* ≤ 0.05). The DMI and TDMI were higher in the 20:80 group than in the other groups (*p* ≤ 0.05). The OMI, CPI, EEI, and TEI were from high to low, 20:80, 60:40, and 80:20 in the F:C groups, respectively (*p* ≤ 0.05). Similarly, CFI, NDFI, and ADFI were from high to low, 80:20, 60:40, and 20:80 in the F:C groups, respectively (*p* ≤ 0.05).

### 3.2. Behavior

During time sampling observations, lying, forage feeding, and rumination behaviors of lambs varied across F:C groups (Table 4). Lying behavior was most prevalent in the 20:80 group, while being least observed in the 80:20 group among lambs (*p* ≤ 0.05). Conversely, forage feeding and rumination behaviors were highest in the 80:20 group and lowest in the 20:80 group (*p* ≤ 0.05). Lying behavior was significantly higher in the 20:80 group than in the other groups, with the lowest rate observed in the 80:20 group (*p* ≤ 0.05). Additionally, while forage feeding, concentrate feeding, rumination, and abnormal stereotypic behaviors varied significantly based on observation day, lying down, roughage consumption, and rumination behaviors showed significant differences based on observation time (*p* ≤ 0.05).

Likewise, the behavior patterns of standing, forage feeding, concentrate feeding, and rumination in goat kids exhibited significant variations across F:C groups (Table 4). Specifically, the 20:80 group displayed a higher frequency of standing behavior compared to the other groups (*p* ≤ 0.05). Forage feeding behavior varied significantly among all groups, with the order being 80:20, 60:40, and 20:80, respectively (*p* ≤ 0.05). Differences in concentrate feeding behavior were found between the 20:80 group and the 80:20 group (*p* ≤ 0.05). Additionally, rumination behavior was observed at a lower rate in the 20:80 group compared to the other groups (*p* ≤ 0.05). In particular, rumination and abnormal stereotypic behavior varied according to observation day, while lying, forage feeding, and rumination behavior varied according to observation time (*p* ≤ 0.05).

In Table 5, the daily frequency for behaviors observed using continuous observation is given per animal. In lambs, bar biting, crib biting, wool biting, rumination, and drinking water behaviors exhibited variations based on F:C groups. As compared with the other groups, the 20:80 group showed greater amounts of bar biting, crib biting, wool biting, and drinking behavior than the other groups (*p* ≤ 0.05). Observation day also affected bucket biting, pawing, social contact, grooming, and rumination behaviors in lambs (*p* ≤ 0.05).

In goat kids, bar biting, crib biting, bucket biting, chain chewing, grooming, rumination, and drinking behaviors exhibited variations across F:C groups (Table 5). There was a higher frequency of bar biting, crib biting, bucket biting, chain chewing, and grooming behaviors in the 20:80 group, but a lower frequency of rumination (*p* ≤ 0.05). Drinking behavior also differed between the 20:80 and 80:20 groups (*p* ≤ 0.05). Furthermore, the observation day had a substantial impact on bucket biting, wool biting, pawing, social contact, and rumination behaviors (*p* ≤ 0.05).

**Figure 2:**
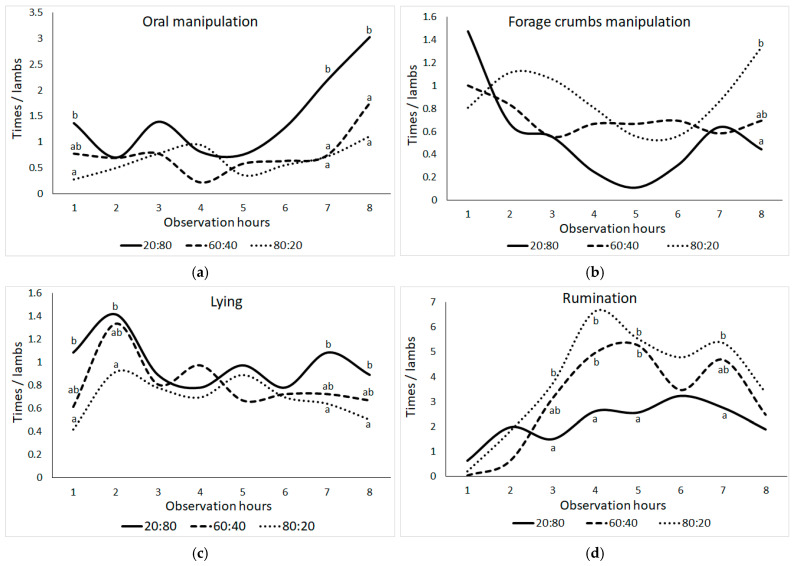
Change in the means of frequencies (times) per animal of (**a**) oral manipulation behavior (bar biting + crib biting + bucket biting + chain chewing + wool biting + ear biting), (**b**) forage crumbs manipulation, (**c**) lying, and (**d**) ruminating behaviors in lambs according to the observation hour (the difference between group means shown with different letters in the same observation hour is statistically significant, *p* ≤ 0.05).

As shown in Figure 2, behavioral frequencies for lambs have changed across different observation hours, including oral manipulation behaviors (bar biting, crib biting, bucket biting, chain chewing, wool biting, and ear biting), forage manipulation behaviors, lying, and ruminating behaviors. In lambs, oral manipulation behavior was significantly higher in the 20:80 group compared to other groups at the first observation hour (except for the 60:40 group), seventh, and eighth observation hours (*p* ≤ 0.05). After 5 h of observation, the 20:80 group showed an increase, while the other groups showed increases after 7 h. At the eighth observation time, there was a significant difference in the 20:80 and 80:20 groups’ manipulation of forage crumbs (*p* ≤ 0.05). After the first hour of observation, the 20:80 group dropped significantly, only to rebound by the fifth hour. In contrast, the manipulation behavior of forage crumbs in the 60:40 group exhibited a consistent level throughout the observation hours. However, the 80:20 group demonstrated an increase in this behavior during the sixth observation hour, reaching its highest point during the eighth observation hour.

Throughout the observation hours, there were persistent tendencies in lying conduct. However, when comparing the first, second, seventh, and eighth observation hours, the 80:20 group had lower frequencies and the 20:80 group had greater frequencies (*p* ≤ 0.05). Rumination behavior varied across F:C groups at the third, fourth, fifth, and seventh observation hours (*p* ≤ 0.05). When compared to the other groups, the 20:80 group’s rumination frequency was most constant between the fourth and fifth observation hours, while the 80:20 group’s rumination frequency was most inconsistent between the third and eighth observation hours (*p* ≤ 0.05).

**Figure 3:**
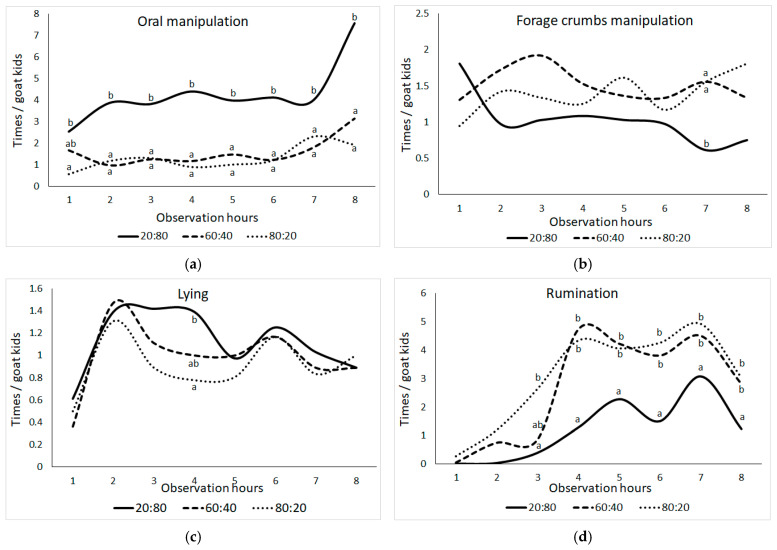
Change in the means of frequencies (times) per animal of (**a**) oral manipulation behavior (bar biting + crib biting + bucket biting + chain chewing + wool biting + ear biting), (**b**) forage crumbs manipulation, (**c**) lying, and (**d**) ruminating behaviors in goat kids according to the observation hour (the difference between group means shown with different letters in the same observation hour is statistically significant, *p* ≤ 0.05).

Figure 3 displays the significant shifts in the frequencies of oral manipulation behaviors observed among goat kids throughout different observation hours, with the 20:80 group standing out in comparison to the others (Figure 3; *p* ≤ 0.05). While the frequencies of oral manipulation behavior exhibited consistency throughout the observation hours in the F:C groups, it is noteworthy to mention the notable increase observed in the 20:80 group during the eighth hour. Furthermore, the 20:80 group’s forage crumbs manipulation behavior was significantly lower than the other groups at the seventh observation time (*p* ≤ 0.05). All F:C groups exhibited similar lying behavior frequencies throughout the day, but by the fourth observation hour, there were noticeable differences between the 20:80 and 80:20 groups (*p* ≤ 0.05). Furthermore, the frequency of rumination behavior varied substantially between F:C groups at the third, fourth, fifth, sixth, seventh, and eighth observation hours (*p* ≤ 0.05). At the third observation hour, both the 20:80 group and the 80:20 group diverged from each other, while at the fourth and fifth observation hours, the 20:80 group demonstrated reduced rumination behavior frequencies compared to the other groups (*p* ≤ 0.05).

### 3.3. Blood Chemicals and Hormone

In lambs, glucose, BUN, CK, and NEFA values showed no significant differences across F:C feed groups (Table 6). Nevertheless, there was a notable disparity in cortisol levels observed across the different groups. Cortisol concentrations of 20:80 group were significantly lower than 80:20 group (*p* ≤ 0.05), but similar to 60:40 group. Furthermore, BUN, CK, and NEFA levels differed statistically from control days (*p* ≤ 0.05).

In goat kids, cortisol hormone levels differed significantly across F:C feed groups (Table 6). Cortisol concentration varied between the 20:80 and 80:20 groups (*p* ≤ 0.05). Additionally, glucose, CK, and NEFA values exhibited significant changes compared to control days in goat kids (*p* ≤ 0.05).

## 4. Discussion

Increases in concentrated feed resulted in greater rates of LW gain and gain rate in both Tahirova lambs and Turkish Saanen goat kids, even though variations in F:C ratios had no discernible effect on the animals’ final LWs. In both lambs and goat kids, higher concentrated feed levels increased DMI, OMI, CPI, EEI, and TEI. However, the overall performance of DMI and FCR was not significantly impacted by these values. Notably, only groups with high concentrated feed showed numerical and proportional increases in live weight values (LW gain in kg and gain rate in %). A 12-week study using F:C feeding ratios of 100:0, 80:20, and 60:40 in Tahalli lambs resulted in significantly higher nutrient and live weight values in the 60:40 group than in the 100:0 group [3]. In Alpine × Beatal crossbred goat kids aged 3–4 months, with F:C ratios of 30:70, 50:50, and 80:20, live weight gain, along with DMI, CPI, and TDNI, was reported to be higher in the 30:70 group than in the other groups [15]. The findings suggest that the consumption of feed and nutrients, along with a low proportion of forage in the daily ration, has a more pronounced influence on behaviors, particularly abnormal stereotypic behaviors, as opposed to performance qualities. Therefore, in this study, the effects of F:C ratios on behavior were discussed in more detail.

One notable observation pertained to the frequency of water intake and drinking behavior. The 80:20 group consumed less water than the other groups because it included more forage and less concentrated feed in the daily diets of the goat kids. Furthermore, increased concentrated feed intake was linked to increased water consumption in both lambs and goat kids. Similar trends were observed in calves, where a decrease in fodder consumption was followed by an increase in drinking time [23]. In general, daily water consumption of 2–3 times the amount of DMI is regarded normal in animals. Excessive water consumption, on the other hand, may be harmful to the organism and animal welfare. In situations of heightened stress, animals may display atypical behavior, including heightened water intake and engaging in manipulative actions towards the water source. It is critical to assess the abnormality level of this increased water consumption (polydipsia) in calves connected to hay presentation [24]. In our study, the high water intake and frequent drinking behavior of groups with a high concentration ratio raises the question of whether this is a normal physiological need or an indication of abnormal stereotypical behavior. Additional research is needed to better understand the effects of this problem on animal welfare.

The F:C feed ratios had a linear effect on forage and concentrate feed intake as well as rumination behavior in lambs and goat kids. An increase in the amount of roughage in the daily diet resulted in an increase in the rate and frequency of roughage consumption and rumination behavior. It is well known that the frequency of rumination increases when daily diets contain more structural carbohydrates like NDF, ADF, and CF [25,26]. In addition, studies have shown that rumination behavior increases as the size of forage particles in the daily ration increases [27]. In contrast, the absence of roughage and a high concentration of concentrated feed in the daily diet decreases rumination behavior, potentially leading to abnormal stereotypic behavior [9,10,28]. Chewing and rumination are crucial for salivation and rumen pH regulation in ruminants. It has been proposed that inadequate saliva secretion in the absence of roughage can result in manipulative oral abnormal stereotypic behaviors [29]. The frequency of oral stereotypic behaviors and rumination behaviors in this study makes it difficult to clearly substitute one for the other when they are combined. There was, however, a greater frequency of abnormal stereotypic behaviors in the goat kids 20:80, characterized by low rumination.

Eating times are lengthened when animals have access to forage or when a significant portion of their diet consists of forage [3,24]. The present study observed that overall eating behaviors, encompassing both forage and concentrate feeding, had a positive correlation with the increased proportion of forage in the daily dietary intake. The assessment of animal levels of satisfaction is frequently determined by increased periods of lying and less periods of standing [2,3]. We found that the greater concentration of feed was strongly connected to the greater prevalence of lying behavior in lambs. Goat kids’ resting behavior was stable across F:C groups, whereas their standing behavior trended upward with increasing feed concentration. These data imply that the F:C ratio in lambs does not cause stress, but rather that lying down behavior is an adaptive reaction to stress. The higher standing behavior ratio in the 20:80 group of goat kids, on the other hand, could be a sign of a chronic type of stress. Furthermore, when the duration per lying activity was compared to the frequency of lying behavior (lying ratio/lying frequency), the 20:80 group of goat kids had a shorter duration per lying behavior. In other words, the 20:80 group’s goat kids offspring spent less time lying down than the other kids.

It has been shown that low forage ratios in goat kids’ diets amplify abnormal stereotypic behaviors compared to lambs when F:C feed ratios are varied. A higher frequency of oral object manipulation was observed in goat kids within the F:C groups, including bar biting, crib biting, bucket biting, and chain chewing compared with lambs. Compared to goat kids, lambs have a slightly longer fleece, which may explain the difference in wool-biting behavior. In particular, goat kids showed high frequency of grooming behavior, a behavior not extensively explored in previous studies. In the 20:80 group, grooming behavior occurred approximately 37.10 ± 4.82 times per animal per day. A study on calves indicated that grooming behavior could become significant stereotypical behavior beyond a certain frequency [24]. Thus, goat kid grooming behavior emerges as an important parameter for assessing animal welfare, if evaluated appropriately.

As the observation hours progressed, goat kids in the 20:80 group demonstrated dramatically more oral manipulation behaviors than goat kids in other groups. A significant increase in oral manipulation behaviors occurred across all F:C groups for lambs and goat kids as evening feeding approached. A decrease in oral stereotypy was observed in lambs in the morning before feeding, while an increase in locomotor stereotypy was observed. It was found that oral stereotypical behaviors stayed the same throughout the day, and pacing behavior significantly decreased after feeding [9]. According to studies on cattle, abnormal stereotypic behaviors are most frequently observed during daylight hours, occurring 0.2–0.9 times per animal per hour [30]. After feeding cattle for 2–4 h, abnormal stereotypic behavior was noted [31]. In the present study, both animal species showed a significant rise in abnormal stereotypic behavior frequency during the final hour of observation in the 20:80 group. As feeding time approaches, this pattern indicates that roughage consumption motivation peaks.

The proportion of forage in lambs’ and goat kids’ daily diets seems to correlate with their cortisol levels. Studies in pigs have shown that fiber consumption reduces cortisol levels [32,33]. In cattle, cortisol levels increased as the NDF content increased in the total mixed ration [34]. Cortisol levels measured significantly lower than those found in goat kids after temporary social isolation tests and after weaning. Stress behaviors and cortisol levels may not always correlate in the same way [21]. Compared to barren environments, lambs in enriched environments showed higher cortisol, NEFA, and CK levels [16]. It is unclear whether coping or stress hypotheses are corroborated by the relationship between cortisol concentration and stereotypic behavior. The relationship is influenced by circadian rhythms, individual variation, and sampling methods [35]. There may be ambiguity due to the stereotypic behavior studied or chronic environmental stress, making it difficult to determine whether lambs and goat kids’ cortisol levels truly reflect stress levels.

## 5. Conclusions

The study showed similar performance and blood parameters (glucose, BUN, NEFA, CK) in Tahirova sheep lambs and Turkish Saanen goat kids, despite differences in their F:C ratios. However, normal and abnormal stereotypic behaviors varied between the groups. Forage feeding and concentrate feeding behaviors aligned with the F:C ratios, while rumination behavior correlated with the roughage ratio in the diet. Lambs exhibited higher frequencies of bar biting, crib biting, and wool-biting behaviors, whereas goat kids displayed more bar biting, crib biting, bucket biting, and chain chewing behaviors in the 20:80 group than other groups. It is worth noting that repetitive grooming behavior in goat kids may turn into abnormal stereotyped behavior, and this should be considered in future studies. To comprehend the relationship between F:C feed ratio, physiological health, metabolic factors, and abnormal stereotypic behavior, further research is necessary. These efforts will significantly enhance our understanding of how such management practices impact animal welfare in the future.

## Figures and Tables

**Table 1:** Chemical composition of forage and concentrate feeds used for feeding.

Feeds	DM	CP	NDF	ADF	ADL	EE	CF	OM	Ash	ME
Alfalfa	923.3	176.4	551.3	319.1	92.8	26.6	226.3	831.4	91.9	2.06
Concentrate	932.5	166.5	326.9	100.6	25.5	32.1	75.1	864.4	68.1	2.88

DM: dry matter, g/kg (as fed basis); CP: crude protein, g/kg DM; NDF: neutral detergent fiber, g/kg DM; ADF: acid detergent fiber, g/kg DM; ADL: acid detergent lignin, g/kg DM; EE: ether extract g/kg DM; CF: crude fiber, g/kg DM; OM: organic matter, g/kg DM; Ash: g/kg; DM. ME: metabolizable energy (Mcal ME/kg DM).

**Table 2:** Least squares mean (LSM), standard error of the mean (SEM), and *p*-values of live weight and feed consumption values in F:C groups in lambs and goat kids.

Animals	Group	20:80	60:40	80:20		
	Items	LSM	LSM	LSM	SEM	*p*
Lambs	Initial LW, kg	33.48	33.76	33.51	1.02	0.9775
	Final LW, kg	37.13	36.81	35.96	1.06	0.7293
	LW gain, kg	3.65 ^a^	3.05 ^ab^	2.45 ^b^	0.35	0.0932
	Gain rate, %	11.08 ^a^	8.97 ^ab^	7.35 ^b^	1.17	0.1130
	DMI, kg	47.10	46.45	46.75	1.50	0.9548
	FCR	14.49	15.98	20.23	2.21	0.1970
Goat kids	Initial LW, kg	19.96	19.78	19.81	1.11	0.9924
	Final LW, kg	21.81	20.16	20.50	1.05	0.5217
	LW gain, kg	1.85 ^a^	0.38 ^b^	0.68 ^b^	0.31	0.0113
	Gain rate, %	9.54 ^a^	2.13 ^b^	3.72 ^b^	1.66	0.0161
	DMI, kg	28.76	26.92	26.30	1.49	0.4947
	FCR	18.20	22.71	17.82	6.75	0.8514

LW: live weight; DMI: the total dry matter intake during the experiment; FCR: feed conversion ratio. The difference among group means shown with different letters on the same line is statistically significant (*p* ≤ 0.05).

**Table 3:** Least squares mean (LSM), standard error of the mean (SEM), and *p* values of nutritional matter intakes and ratio in F:C feed groups in lambs and goat kids.

Animals	Group	20:80	60:40	80:20		
	Items	LSM	LSM	LSM	SEM	*p* Value
Lambs	F:C, %	19.97 ^a^	57.43 ^b^	78.29 ^c^	0.25	<0.0001
	WI, kg/day	4.08	3.92	3.88	0.23	0.8081
	DMI, g/day	1309.7	1291.7	1299.9	10.23	0.4580
	TDMI (g/kg W ^0.75^/d)	541.7	534.8	538.9	4.05	0.4761
	OMI, g/day	1123.2 ^a^	1092.3 ^b^	1089.9 ^b^	8.68	0.0105
	CPI, g/day	241.9 ^a^	243.9 ^ab^	248.7 ^b^	1.92	0.0381
	EEI, g/day	40.6 ^a^	37.4 ^b^	36.1 ^c^	0.30	<0.0001
	CFI, g/day	139.4 ^a^	208.1 ^b^	252.0 ^c^	1.70	<0.0001
	NDFI, g/day	489.1 ^a^	587.1 ^b^	654.1 ^c^	4.63	<0.0001
	ADFI, g/day	191.1 ^a^	290.5 ^b^	353.9 ^c^	2.39	<0.0001
	TEI, ME/day	3.55 ^a^	3.12 ^b^	2.91 ^c^	0.02	<0.0001
Goat kids	F:C, %	19.51 ^a^	58.14 ^b^	78.25 ^c^	0.19	<0.0001
	WI, kg/day	3.31 ^a^	3.26 ^a^	2.33 ^b^	0.28	0.0472
	DMI, g/day	803.2 ^a^	748.6 ^b^	731.4 ^b^	8.44	<0.0001
	TDMI (g/kg W ^0.75^/d)	376.1 ^a^	356.0 ^b^	346.7 ^b^	3.46	<0.0001
	OMI, g/day	689.1 ^a^	632.7 ^b^	613.2 ^c^	7.15	<0.0001
	CPI, g/day	148.2 ^a^	141.5 ^b^	139.9 ^c^	1.58	0.0005
	EEI, g/day	24.9 ^a^	21.6 ^b^	20.3 ^c^	0.24	<0.0001
	CFI, g/day	83.9 ^a^	122.3 ^b^	141.8 ^c^	1.35	<0.0001
	NDFI, g/day	297.6 ^a^	342.8 ^b^	368.0 ^c^	3.78	<0.0001
	ADFI, g/day	114.9 ^a^	170.8 ^b^	199.1 ^c^	1.90	<0.0001
	TEI, ME/day	2.18 ^a^	1.80 ^b^	1.64 ^c^	0.02	<0.0001

F:C realized forage-to-concentrate ratio; WI: water intake; DMI: total dry matter intake; TDMI: g/kg W 0.75/d, gram per kilogram of metabolic body weight per day; OMI: total organic matter intake; CPI: total crude protein intake; EEI: total ether extract intake; CFI: total crude fiber intake; NDFI: total neutral detergent fiber intake; ADFI: total acid detergent fiber intake; TEI: total metabolizable energy intake. The difference among group means shown with different letters on the same line is statistically significant (*p* ≤ 0.05).

**Table 4:** Mean ± standard error and *p* values of behaviors determined by time sampling method in lambs and goat kids according to F:C feed groups *.

Animals	Behaviors %	Group			
		20:80	60:40	80:20	*p* Value
Lambs	Lying	59.03 ± 1.29 ^a^	50.97 ± 1.31 ^b^	39.44 ± 1.28 ^c^	0.0154
	Standing	11.11 ± 0.82	8.33 ± 0.72	7.29 ± 0.68	0.4689
	Forage feeding	5.83 ± 0.61 ^a^	16.94 ± 0.98 ^b^	22.29 ± 1.09 ^c^	0.0036
	Concentrate feeding	7.50 ± 0.69	2.29 ± 0.39	3.89 ± 0.50	0.3680
	Rumination	10.07 ± 0.79 ^a^	16.25 ± 0.97 ^b^	22.99 ± 1.10 ^c^	0.0059
	Moving	3.26 ± 0.46	2.43 ± 0.40	1.88 ± 0.35	0.2138
	Abnormal stereotypic	3.19 ± 0.46	2.78 ± 0.43	2.22 ± 0.38	0.5686
Goat kids	Lying	42.08 ± 1.30	45.42 ± 1.31	41.46 ± 1.29	0.5534
	Standing	19.79 ± 1.05 ^a^	11.11 ± 0.82 ^b^	10.83 ± 0.81 ^b^	0.0172
	Forage feeding	8.47 ± 0.73 ^a^	14.31 ± 0.92 ^b^	18.89 ± 1.03 ^c^	0.0073
	Concentrate feeding	8.19 ± 0.72 ^a^	5.69 ± 0.61 ^ab^	4.51 ± 0.54 ^b^	0.0446
	Rumination	6.11 ± 0.63 ^a^	13.13 ± 0.89 ^b^	15.28 ± 0.94 ^b^	0.0091
	Moving	5.90 ± 0.62	4.17 ± 0.52	3.82 ± 0.50	0.2552
	Abnormal stereotypic	9.44 ± 0.77	6.18 ± 0.63	5.21 ± 0.58	0.2822

* Statistical analyses were performed according to binomial distribution. The difference between group means shown with different letters on the same line is statistically significant (*p* ≤ 0.05).

**Table 5:** Mean ± standard error and *p* values of behaviors determined by continuous sampling method in lambs and goat kids according to F:C feed groups *.

Animals	Behaviors, Times	Group (G)			
		20:80	60:40	80:20	*p*
Lambs	Bar bating	5.86 ± 1.36 ^a^	3.06 ± 0.57 ^b^	2.83 ± 0.84 ^b^	0.0430
	Crib biting	1.56 ± 0.35 ^a^	0.80 ± 0.25 ^b^	0.60 ± 0.18 ^b^	0.0263
	Bucket biting	2.73 ± 0.82	2.36 ± 0.54	2.43 ± 0.64	0.9741
	Wool biting	2.76 ± 1.01 ^a^	1.03 ± 0.51 ^b^	0.30 ± 0.14 ^b^	0.0235
	Chain chewing	0.86 ± 0.63	0.16 ± 0.08	0.13 ± 0.09	0.3715
	Forage crumbs manipulation	5.33 ± 0.95	6.83 ± 1.41	8.50 ± 1.36	0.2088
	Pawing	2.46 ± 0.97	1.50 ± 0.50	1.63 ± 0.80	0.6569
	Social contact	2.56 ± 0.58	2.03 ± 0.37	1.60 ± 0.48	0.2272
	Bipedal stance	1.33 ± 0.49	1.20 ± 0.33	2.46 ± 0.83	0.3330
	Grooming	12.10 ± 1.41	8.96 ± 1.11	9.10 ± 1.29	0.1137
	Lying	9.46 ± 0.61 ^a^	7.80 ± 0.54 ^b^	6.63 ± 0.65 ^b^	0.0026
	Rumination	20.56 ± 2.03 ^a^	29.60 ± 3.44 ^b^	37.66 ± 3.40 ^c^	0.0004
	Drinking water	6.26 ± 0.97 ^a^	3.13 ± 0.54 ^b^	3.80 ± 0.71 ^b^	0.0034
Goat kids	Bar biting	14.83 ± 2.66 ^a^	5.00 ± 0.99 ^b^	4.76 ± 0.88 ^b^	<0.0001
	Crib biting	8.83 ± 2.17 ^a^	3.43 ± 0.59 ^b^	2.73 ± 0.52 ^b^	0.0005
	Bucket biting	6.50 ± 1.44 ^a^	3.56 ± 0.67 ^b^	2.43 ± 0.51 ^b^	0.0121
	Wool biting	1.23 ± 0.40	2.36 ± 0.71	1.76 ± 0.65	0.3582
	Chain chewing	9.66 ± 2.77 ^a^	0.86 ± 0.37 ^b^	0.70 ± 0.28 ^b^	<0.0001
	Forage crumbs manipulation	9.90 ± 1.13	14.46 ± 1.76	13.30 ± 2.19	0.2422
	Pawing	1.33 ± 0.42	0.83 ± 0.32	1.73 ± 0.93	0.6284
	Social contact	4.96 ± 1.23	2.76 ± 0.55	2.76 ± 0.61	0.1330
	Bipedal stance	16.96 ± 2.46	18.00 ± 2.21	17.13 ± 1.50	0.8284
	Grooming	37.10 ± 4.82 ^a^	33.76 ± 3.42 ^a^	23.16 ± 2.61 ^b^	0.0286
	Lying	10.73 ± 0.90	9.46 ± 1.05	8.73 ± 0.87	0.2406
	Rumination	11.73 ± 1.71 ^a^	26.06 ± 3.16 ^b^	29.63 ± 2.67 ^b^	<0.0001
	Drinking water	7.53 ± 1.30 ^a^	5.00 ± 0.48 ^ab^	4.36 ± 0.68 ^b^	0.0530

* Logarithmic (y + 10) transformation was applied to the data. The difference between group means shown with different letters on the same line is statistically significant (*p* ≤ 0.05).

**Table 6:** Least square means, standard errors mean (SEM), and *p* values of biochemical traits and cortisol hormone by F:C feed groups in lambs and goat kids.

Animals		Group				
	Items	20:80	60:40	80:20	SEM	*p* Value
Lambs	Glucose, mg/dL	63.81	66.20	62.18	3.74	0.7494
	BUN, mg/dL	16.19	15.97	15.97	0.14	0.4869
	CK, U/L	113.92	117.58	98.14	13.27	0.5425
	NEFA, mmol/L	0.22	0.22	0.19	0.02	0.4182
	Cortisol, ng/mL	17.93 ^a^	19.06 ^ab^	22.63 ^b^	1.22	0.0477
Goat kids	Glucose mg/dL	71.58	75.18	72.89	2.11	0.3487
	BUN, mg/dL	17.28	17.70	17.17	0.22	0.3967
	CK, U/L	78.42	77.96	81.36	8.93	0.8747
	NEFA, mmol/L	0.21	0.24	0.22	0.01	0.0681
	Cortisol, ng/mL	9.82 ^a^	11.56 ^ab^	15.05 ^b^	1.59	0.0169

BUN: blood urea nitrogen, CK: creatine kinase; NEFA: non-esterified fatty acid. The difference between group means shown with different letters on the same line is statistically significant (*p* ≤ 0.05).

## Data Availability

The data presented in this study are available upon reasonable request from the author.
